# Treatment of Warts

**Published:** 1846-12

**Authors:** 


*Treatment of Warts.*—The hydrochlorate of ammonia dissolved in water, and the hydrochlorate of lime are the most certain means of destroying them; the process, however, in both instances is very slow, and demands perseverance, for if discontinued before the proper time, no advantage is derived.—*Eisenberg's Advice on the Hand.—Dublin Medical Press.—Ibid.*

